# SARS-CoV-2 infection complicated by acute longitudinally extensive transverse myelitis and pulmonary embolism: a case report

**DOI:** 10.3389/fcvm.2026.1811261

**Published:** 2026-05-01

**Authors:** Gulinazi Rouzi, Kamiernisa Wupuer, Ailiyaer Yasheng, Yang Liu, Reziya Apaer, Wumanjiang Yidayi, Taxi Wumiti, Pu Miao, Muhetaer Munire, Zhijian Li, Aizezi Aihemaitiniyazi

**Affiliations:** 1 Department of Rehabilitation, Uygur Medical Hospital of Xinjiang Uygur Autonomous Region (The Second People's Hospital of Xinjiang Uygur Autonomous Region), Urumqi, China; 2 Key Laboratory of Evidence-Based and Translation, Xinjiang Hospital, Preparation of Traditional Chinese Medicine, Urumqi, China

**Keywords:** a case report, acute myelitis, longitudinally extensive transverse myelitis, pulmonary embolism, SARS-CoV-2

## Abstract

Coronavirus disease 2019 (COVID-19) presents with a wide spectrum of clinical manifestations. While primarily affecting the respiratory system, neurological involvement and thromboembolic complications have also been documented. This report describes a rare case of a 25-year-old male who developed acute longitudinally extensive transverse myelitis (LETM) complicated by pulmonary embolism (PE) and deep vein thrombosis (DVT) of the lower extremities following SARS-CoV-2 infection. The patient presented with acute chest tightness and pain, followed by high fever, which rapidly progressed to quadriplegia and sensory deficits. Magnetic resonance imaging (MRI) revealed abnormal signal intensity in the spinal cord from the C4 to T1 levels. Computed tomography pulmonary angiography (CTPA) confirmed PE, and laboratory tests were positive for SARS-CoV-2 nucleic acid. Although aquaporin-4 (AQP4), myelin oligodendrocyte glycoprotein (MOG), and glial fibrillary acidic protein (GFAP) antibodies were negative, cerebrospinal fluid analysis indicated inflammatory changes. Partial clinical improvement was achieved following treatment with high-dose corticosteroid pulse therapy, anticoagulation, and antiviral agents. This case highlights the potential of COVID-19 to simultaneously trigger neurological and vascular complications, underscoring the complexity of multi-system involvement by the virus. The underlying pathophysiological mechanisms involving potential “virus-immune-thrombosis” interactions are discussed in depth, providing important insights for clinicians to recognize and manage such complex cases.

## 1 Introduction

The outbreak of Coronavirus Disease 2019 (COVID-19) at the end of 2019 evolved into a global public health crisis. Although it is currently under control, sporadic cases continue to occur. The clinical manifestations of COVID-19 are complex and diverse, potentially leading to fever, cough, diarrhea, generalized myalgia, severe respiratory diseases, and severe respiratory failure that may progress to acute respiratory distress syndrome (ARDS) (1). According to existing literature, the clinical manifestations of this condition extend well beyond acute respiratory symptoms; as a systemic disease, it can affect multiple organ systems, including the cardiovascular, nervous, and coagulation systems (2). Notably, thromboembolic events and neurological complications are significant contributors to disease progression, poor prognosis, and mortality in patients, garnering increasing attention from both clinical practice and scientific research. It is worth noting that these two severe complications, which appear to belong to different systems, may in fact stem from a common pathophysiological basis: SARS-CoV-2 places the vascular system and the central nervous system on the same axis of damage through angiotensin-converting enzyme 2 (ACE2) receptor-mediated endothelial injury, excessive inflammatory responses (such as cytokine storm), and the resulting immune thrombogenesis and neuroimmune cross-reactions. Therefore, when both occur simultaneously in the same patient, they should not be regarded as a coincidental overlap; rather, one should be vigilant for the possibility of underlying “virus-immune-thrombotic” interactions.

Among thromboembolic complications, pulmonary embolism (PE) is particularly prominent. Numerous large-scale epidemiological studies have demonstrated a significantly elevated risk of PE in patients with COVID-19. A study conducted in the United States revealed that during the ancestral variant period, 18.3% of COVID-19-positive patients underwent computed tomography pulmonary angiography (CTPA); this percentage remained at 18.3% of COVID-19-positive patients underwent CTPA; this proportion was 18.3% during the Delta variant period and 17.3% during the Omicron variant period. The incidence of PE among all COVID-19-positive emergency department visits was 1.94% during the Delta period and 1.6% during the Omicron period. Compared to the ancestral period, the incidence of PE among all COVID-19-positive emergency department visits was higher during the Delta period (3). In a Swedish case-crossover study, a risk ratio for DVT was 4.98 (4.96 to 5.01), for PE was 33.05 (32.8 to 33.3), and for bleeding was 1.88 (1.71 to 2.07) within 1 to 30 days after COVID-19 infection (4). An investigation across eight hospitals in the Netherlands found that the adjusted cumulative incidence of all thrombotic complications was 12%, 16%, and 21% after 10, 20, and 30 days, respectively. The patient demographics were consistent across the two pandemic waves (5). Notably, the risk of PE and DVT is also increased even in individuals with mild infection. A case report described a 76-year-old patient who contracted a mild case of COVID-19 in September 2021 and developed a completely spontaneous popliteal hematoma four weeks later, followed by deep vein thrombosis (DVT). Treatment with low-molecular-weight heparin (LMWH) was initiated, but the patient subsequently developed extensive subxiphoid and calf hematomas, leading to moderate post-hemorrhagic anemia and acute kidney injury (6). Clinical data indicate that the reported incidence of PE among hospitalized COVID-19 patients varies across studies, and this proportion is significantly higher in critically ill patients admitted to the intensive care unit (ICU) (7). The pathophysiological mechanisms underlying these complications are thought to be intricately linked to systemic inflammatory response, vascular endothelial injury, and unique coagulation dysfunction triggered by SARS-CoV-2 infection.

Concurrently, neurological complications associated with COVID-19 have been frequently reported, encompassing a wide spectrum from peripheral neuropathies to central nervous system demyelinating diseases. Among these, acute transverse myelitis (ATM) is a rare complication of COVID-19, occurring at a rate of less than 0.1%, yet it can result in significant neurological impairments (8). The annual incidence of transverse myelitis itself is extremely low. Existing clinical observations and case reports indicate that SARS-CoV-2 infection can trigger ATM, which may manifest during the acute phase of infection or within weeks after recovery. Patients may present with an acute or subacute onset of limb paralysis, a sensory level, and bladder and bowel dysfunction. Radiologically, some cases may exhibit “long-segment” lesions involving multiple spinal cord segments. Its pathophysiology is complex and may be related to direct viral invasion of the nervous system, post-infectious autoimmune responses, or inflammatory damage caused by a subsequent cytokine storm. One literature report documented a patient from Panama who developed ATM following SARS-CoV-2 infection and provided a comprehensive clinical review of 43 cases of COVID-19-associated ATM from 21 countries between March 2020 and January 2021. Furthermore, during the clinical trial of the ChAdOx1 nCoV-19 (AZD1222) vaccine, three cases of ATM were reported as serious adverse events (9).

Our case report aims to present a detailed account of a complex case involving concurrent acute long-segment transverse myelitis and pulmonary embolism following COVID-19. It seeks to provide a comprehensive discussion on the clinical characteristics, diagnostic challenges, therapeutic dilemmas, and potential underlying mechanisms. The objective is to heighten clinicians' awareness of such critical comorbidities and to offer a new perspective for understanding the long-term multi-system complications associated with COVID-19.

## 2 Case description

### 2.1 Basic information

A 25-year-old male patient, from Xinjiang Uygur Autonomous Region, China, was admitted to a hospital based in Urumqi, on May 18, 2025, presenting with symptoms of chest tightness, chest pain accompanied by weakness and numbness of the limbs for more than one day. On May 16, 2025, the patient experienced a sudden onset of needle-pricking pain in the right anterior chest region without an obvious precipitating factor. The pain lasted for 5–6 min, then shifted to the left side with the same character, persisting for approximately 2–3 min. This was accompanied by severe vomiting, with vomitus consisting of food residue. The chest pain was slightly alleviated after vomiting. At that time, the patient did not exhibit symptoms such as cough, expectoration, fever, chills, or convulsions. Upon admission, his body temperature was 36.8 °C. Subsequently, weakness and numbness in both upper limbs developed, manifesting as an inability to lift both arms. This rapidly progressed to sensory loss and weakness in both lower limbs, resulting in an inability to walk. No dysphagia, choking on water, incontinence of urine or feces, or disturbance of consciousness was reported. In response to the acute symptoms, the patient was transported to the emergency department.

The patient had a prior history of good health, with no history of hypertension, diabetes, or cerebrovascular disease. In August 2024, he underwent surgical intervention for an inguinal hernia. He had a smoking history for three years, averaging four cigarettes per day. No history of drug or food allergies was noticed.

### 2.2 Clinical course and treatment history

#### 2.2.1 Acute phase

##### 2.2.1.1 Admission physical examination

The patient vital signs were as follows: body temperature was 36.8 °C; pulse rate was 75 beats per minute; respiratory rate was 18 breaths per minute; and blood pressure was 87/50 mmHg. The patient was conscious but appeared listless, maintained an autonomous body position and was cooperative during the examination. Both pupils were equal, round, with a diameter of 3 mm, and exhibited prompt light reflexes. Breath sounds were clear bilaterally with no audible dry or moist rales. The heart rate was 75 beats per minute with a regular rhythm, and no murmurs were detected over any valve area. The abdomen was flat, without tenderness or rebound tenderness.

Neurological Examination: Speech was fluent. Memory, calculation ability, and orientation were intact. Cranial nerve examination revealed no abnormalities. The morphology of the limb muscles was normal. Muscle strength assessment was as follows: bilateral shoulder flexion and abduction, grade 3; elbow flexion and extension, grade 3; wrist and finger joint movements, grade 0. For the lower limbs: bilateral hip flexion, grade 0; knee flexion and extension, grade 0; ankle and toe joint movements, grade 0. Sensory examination revealed: diminished sensation in a glove-like distribution over the right upper limb; diminished pain sensation below the line connecting the sternal angle and the nipple (approximately at the T4 dermatome level); and complete loss of pain sensation below the T5 dermatome level. Light touch and deep sensation were preserved. Muscle tone in all four limbs was normal. Tendon reflexes were present. Pathological signs were not elicited. Meningeal signs were negative.

##### 2.2.1.2 Auxiliary examinations

###### 2.2.1.2.1 Laboratory investigations

**Figure 1:**
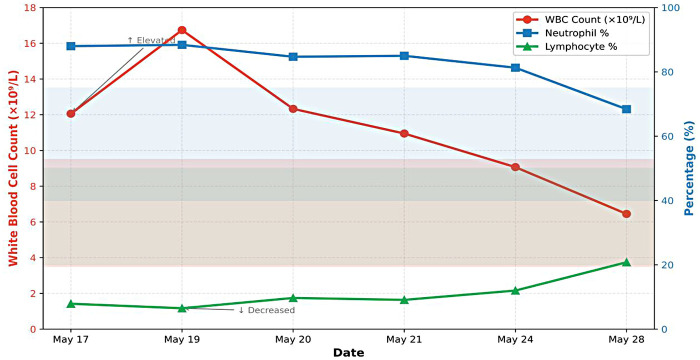
Longitudinal changes in complete blood count parameters.

The blood test results indicated that the percentages of white blood cells and neutrophils reached their peak on May 19th, and then began to decline steadily (Figure 1).

**Figure 2:**
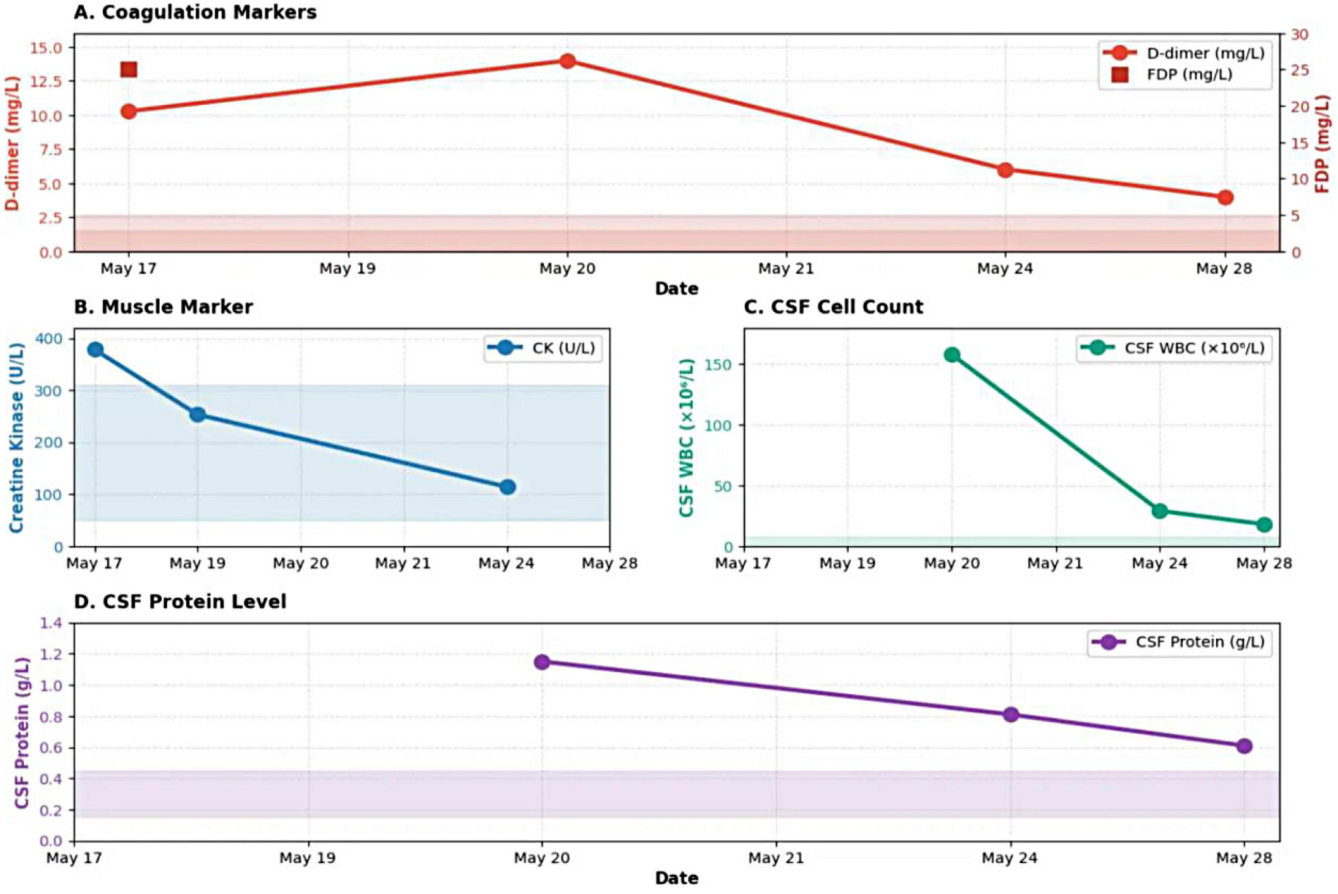
Longitudinal changes in laboratory markers. **(A)** Coagulation Markers. **(B)** Muscle Marker (CK). **(C)** CSF Cell Count. **(D)** CSF Protein Level.

The D-dimer level rose to an extremely high value of 13.98 mg/L on May 20th. After treatment, it steadily decreased (Figure 2A). The Creatine Kinase (CK) result reached its peak on May 17th, and then steadily decreased after treatment (Figure 2B). The cerebrospinal fluid test results showed that on May 20th, the white blood cell and protein levels increased, suggesting an inflammatory process in the central nervous system (protein 1.15 g/L, white blood cells 158 × 10^6^/L) (Figures 2C,D). After treatment, these levels steadily decreased.

Cerebrospinal Fluid (CSF) Analysis (May 19, 2025): Normal opening pressure. White blood cell count: 6.0 × 10^6^/L (mononuclear cells 50%, polymorphonuclear cells 50%). Protein: 0.79 g/L. Glucose and chloride levels were within normal ranges. Staining of the CSF smear revealed no bacteria, acid-fast bacilli, or Cryptococcus neoformans

Autoimmune Antibody Testing: Serum antibodies against aquaporin-4 (AQP4-IgG), myelin oligodendrocyte glycoprotein (MOG-IgG), and glial fibrillary acidic protein (GFAP) were all negative. Other Laboratory Findings: Urine protein (2+); elevated uric acid (544.78 μmol/L); elevated lactate (3.18 mmol/L); elevated triglycerides (3.49 mmol/L); elevated serum alanine aminotransferase (52.42 U/L). A SARS-CoV-2 nucleic acid test was positive on May 27, 2025 (Ct values: ORF1ab gene 37.61, N gene 38.53).

The patient's COVID-19 nucleic acid (ORF-L) test result on May 27, 2025: 37.61; COVID-19 nucleic acid (N gene) test result: 38.53; Test method: Real-time fluorescent PCR; concurrently tested for Legionella pneumophila antibodies (serum type 1 IgM antibodies), Mycoplasma pneumoniae serology (IgM antibodies), Rickettsia serology (Q fever rickettsia), Chlamydia pneumoniae IgM antibodies, adenovirus antibody assay (IgM antibodies), respiratory syncytial virus antibody assay (IgM antibodies), serological diagnosis of influenza A virus (IgM antibody), serological diagnosis of influenza B virus (IgM antibody), and parainfluenza virus antibody testing (IgM antibodies for types 1, 2, and 3), all of which were negative; concurrent general bacterial culture over 48 h showed no bacterial growth. These tests ruled out other bacterial and viral pathogens, serving as a differential diagnosis.

###### 2.2.1.2.2 Imaging examinations

**Figure 3:**
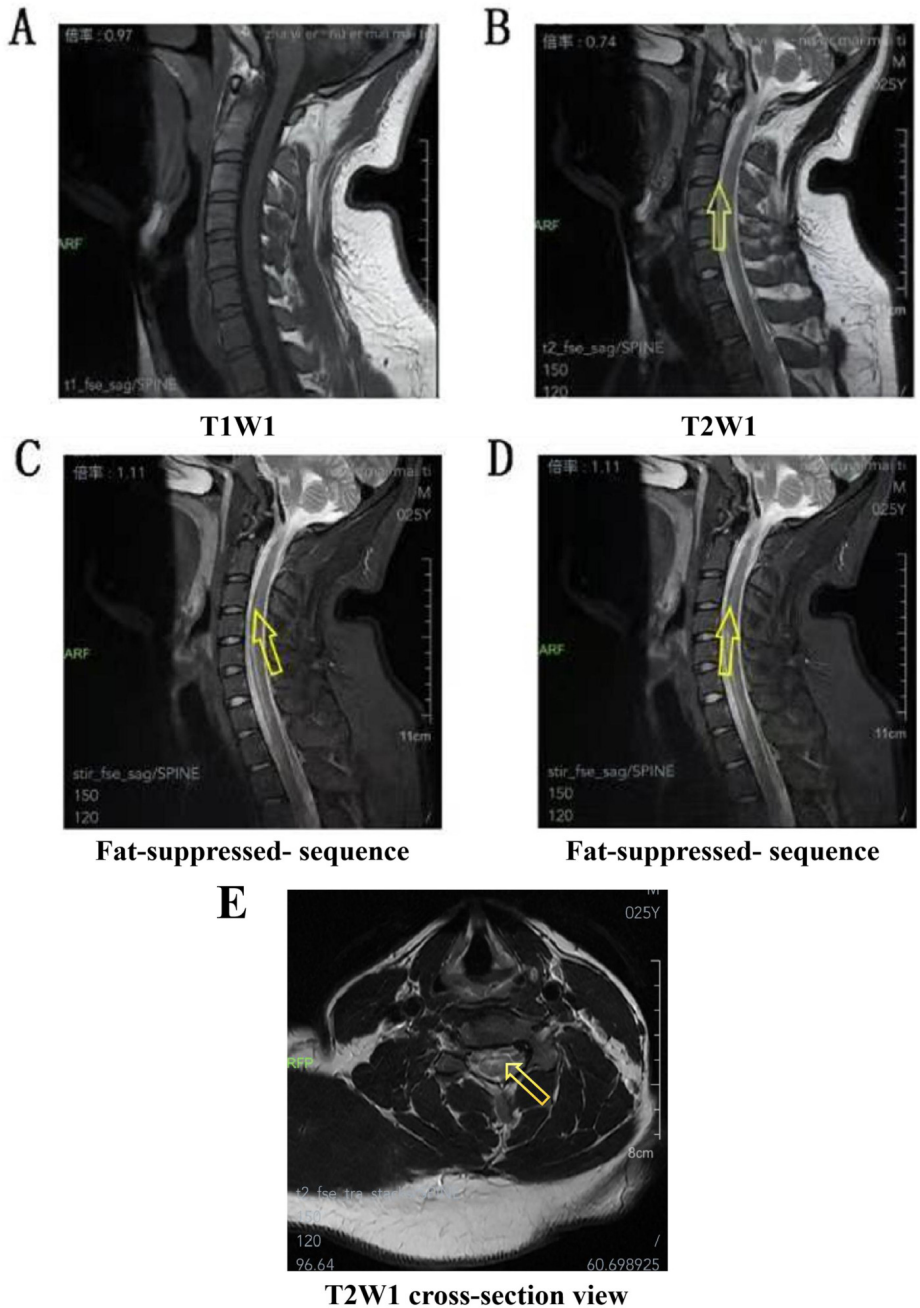
Spinal cord MRI. **(A)** No abnormal signals were observed in T1WI. **(B)** On T2WI, abnormal signals can be observed at the anterior margin of the spinal cord at the level of C4-C1 vertebrae. **(C,D)** Abnormal signals at the anterior margin of the spinal cord at the level of cervical 4 to thoracic 1 vertebra can be observed in the fat-suppression sequence. **(E)** On the T2WI sagittal view, abnormal signals in the spinal cord at the level of the C4-C5 intervertebral disc.

Spinal Cord MRI (May 18, 2025): An abnormal signal was observed along the anterior margin of the spinal cord at the level of the C4 to T1 vertebral bodies, suggestive of possible myelitis (Figure 3). No significant abnormalities were detected on the plain scan of the thoracic spine.

**Figure 4:**
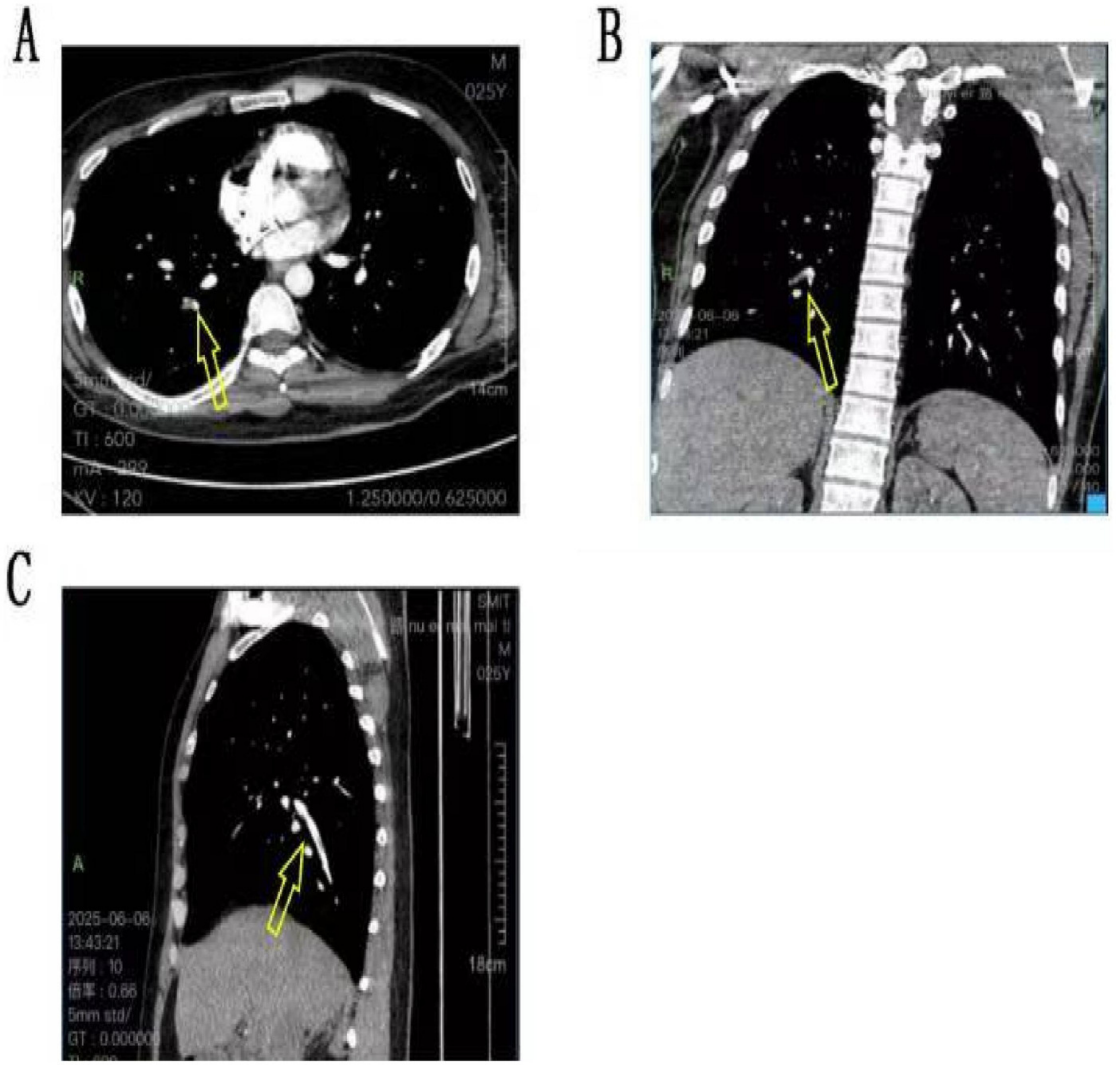
Combined pulmonary artery CTA. **(A–C)** Contrast agent filling in the pulmonary artery lumen is not uniform (indicated by arrows) that suggesting a possible localized pulmonary embolism.

Combined Pulmonary Artery CTA (May 20, 2025):

1. Scattered exudative opacities and linear shadows were noted in the lower lobes of both lungs.
2. A small amount of pleural effusion was present bilaterally, accompanied by minimal subsegmental atelectasis in the lower lobes of both lungs.
3. Computed tomography pulmonary angiography (CTPA) revealed uneven filling of the contrast agent within the lumens of the lingular and anterior segmental arteries of the left upper lobe, raising the possibility of localized pulmonary embolism (Figure 4).

Other examinations: On May 17th, the head MRI showed that the sulci of both cerebral hemispheres were slightly widened; the cisterna magna cavity. The lower extremity venous ultrasound indicated thrombosis in the posterior tibial vein on the left side. No obvious abnormalities were found in the cardiac ultrasound.

##### 2.2.1.3 Acute phase treatment

Immunomodulatory Therapy: Methylprednisolone sodium succinate was administered intravenously at a dose of 500 mg once daily, commencing on May 20. After three consecutive days, the dose was reduced to 240 mg once daily via intravenous drip on May 24. This regimen was maintained for 3 days, after which the dose was further reduced to 120 mg once daily on May 27. The patient was discharged on May 28 and subsequently received injections at an external hospital for 3 days three days, as per medical advice. On May 31, the dosage was adjusted to 80 mg once daily via intravenous drip. The therapy was then switched to oral prednisone at a dosage of 60 mg (12 tablets) once daily, with a planned reduction of one tablet per week.

Anticoagulation Therapy: Enoxaparin sodium was administered via subcutaneous injection at a volume of 0.4 mL every 12 h for the management of pulmonary embolism and deep vein thrombosis (DVT).

Antiviral Therapy: A combination pack of Simnotrelvir Tablets/Ritonavir Tablets was administered orally at a dosage of 300 mg twice daily, initiated on May 27, following the confirmation of a positive COVID-19 test result.

Symptomatic and Supportive Treatment: Mecobalamin and vitamin B1 were administered for neurotrophic support. Potassium and calcium supplementation, along with acid suppression and gastric protection, were provided to prevent corticosteroid-related side effects. Rehabilitation training was implemented to prevent complications.

#### 2.2.2 Course of the disease

The patient was admitted at 15:00 on May 18. Following admission, the patient's condition deteriorated rapidly, characterized by the swift onset of fever, quadriplegia, and sensory disturbances. Based on imaging findings and laboratory tests, a diagnosis of acute myelitis, pulmonary embolism, and SARS-CoV-2 infection was established.

Upon admission, the patient's body temperature was 36.8 °C which increased to 39 °C by 20:00 and further to 39.2 °C by 23:00. Temperature reduction was achieved through physical cooling measures and an intramuscular injection of 2 mL of Antondine. However, over the subsequent days, a fluctuating pattern of temperature elevation followed by reduction after medication was observed. Throughout the hospitalization period, the body temperature fluctuated between 36.2 °C and 39.2 °C, with the highest recorded temperature of 38.7 °C on May 26. Body temperature normalized on May 27 without subsequent recurrence. Recovery of limb muscle strength was slow, and sensory disturbances persisted. Subsequently, the patient developed urinary retention, necessitating indwelling catheterization. Thrombosis markers, including D-dimer and fibrin degradation products (FDP), gradually decreased (Figure 2A), indicating the effectiveness of anticoagulant therapy. Following a three-day regimen of pulse steroid therapy, a modest improvement in proximal upper limb muscle strength was noted (shoulder flexion and abduction muscle strength maintained at grade 3). Lower limb muscle strength recovered from grade 0 to grade 1. However, fine motor control of the wrist and fingers, as well as ambulatory function, remained unrecovered.

During hospitalization, significant complications arose, including neurogenic bladder: Required indwelling urinary catheterization and bladder function training. Autonomic dysfunction: Manifested as anhidrosis, absence of pain sensation, and dry, desquamating skin below the level of the spinal lesion.

Laboratory analyses indicated that the patient was in the acute fulminant phase of the disease from May 17 to 20, characterized by concurrent systemic inflammation and a hypercoagulable state. The white blood cell count and neutrophil percentage peaked (Figure 1), which was consistent with the clinical presentation of acute-onset severe chest pain and rapidly progressing paralysis, indicative of a robust immune system activation. Furthermore, the D-dimer level escalated to a notably high value of 13.98 mg/L (Figure 2A), providing direct laboratory evidence for the simultaneous occurrence of spinal microvascular injury, deep vein thrombosis (DVT) in the lower extremities, and pulmonary embolism (PE). This supports the core pathophysiology that a “cytokine storm leads to endothelial injury and a hypercoagulable state” (10).

Cerebrospinal fluid analysis revealed lymphocytic pleocytosis and elevated protein levels (protein 1.15 g/L, white blood cells 158 × 10^6^/L) (Figure 2C), suggesting an inflammatory process within the central nervous system (CNS). These findings clearly indicate inflammatory changes in the CNS and serve as key evidence for the diagnosis of acute myelitis.

Intervention and Transition Period (May 21–28): Response to Treatment. Following the commencement of high-dose methylprednisolone pulse therapy and enoxaparin anticoagulation on May 19, all key parameters exhibited a favorable improvement. Systemic inflammatory markers, including white blood cell count (WBC) and neutrophil percentage (NEUT%), began to decline steadily (Figure 1). The hypercoagulability marker (D-dimer) demonstrated a clear and rapid downward trend, confirming the effective control of thrombotic events by anticoagulant therapy.

Central nervous system inflammatory indicators, such as cerebrospinal fluid white blood cell count (CSF-WBC) and cerebrospinal fluid protein levels (CSF-Protein), decreased significantly (Figure 2D), demonstrating that corticosteroid therapy effectively suppressed the immune response within the spinal cord. Although levels had not normalized, the downward trend indicated that the disease was transitioning from the active phase to a remission phase. A positive SARS-CoV-2 nucleic acid test result was obtained on May 27 (ORF1ab gene Ct value 37.61, N gene Ct value 38.53), providing etiological evidence. Despite the high Ct values (suggesting low viral load or a later disease stage), this finding clarified the infectious background and is essential for diagnosing “COVID-19-associated complications” (11).

#### 2.2.3 Recovery period

Upon discharge, the patient vital signs were recorded as follows: body temperature was 36.9 °C, pulse rate was 78 beats per minute, and respiratory rate was 18 breaths per minute. Neurological assessment indicated muscle strength in the shoulder girdle of the upper limbs was graded at 3, while distal muscle strength was graded at 0; muscle strength in the lower limbs was graded at 1. Sensory examination revealed hypalgesia below the T4 dermatome and analgesia below the T5 dermatome. The discharge plan included the following: The patient was discharged on May 28. In accordance with medical advice, intravenous methylprednisolone was administered at an external facility for three days, concluding on May 31, after which the regimen was adjusted to 80 mg once daily. Subsequently, the treatment was transitioned to oral prednisone, administered as 12 tablets (60 mg) once daily, with a planned reduction of one tablet per week. Enoxaparin sodium was prescribed for anticoagulation therapy. Regular follow-up appointments in the neurology department and participation in rehabilitation training were advised.

### 2.3 Prognosis and follow-up

The patient subsequently participated in two rehabilitation therapies at our hospital: from September 2 to September 21, 2025, and from October 26 to November 15, 2025. At the conclusion of the second rehabilitation course, the patient's status was as follows: Hypalgesia to pinprick was observed below the T6 level, with normal light touch but analgesia noted below T7. Deep sensation in all four limbs was normal. Light touch sensation was preserved in the saddle area, and the bulbocavernosus reflex was positive. Muscle strength was graded as follows: bilateral elbow flexors, grade 5; elbow extensors, grade 4-; wrist extensors, grade 3; bilateral hip flexors, grade 3; quadriceps, grade 3; tibialis anterior, grade 2-. Sitting balance was insufficient at grade 3, and standing balance was insufficient at grade 1. Following pelvic floor rehabilitation training, the indwelling urinary catheter was removed, and intermittent catheterization was implemented. The prognosis of myelitis is dependent on the severity of the lesion, the timeliness of treatment, and the presence of complications. Long-term management should focus on: neurological function recovery and rehabilitation training; prevention of myelitis-related complications; long-term management of thromboembolic risk; and monitoring for corticosteroid-related side effects.

## 3 Discussion

This case report describes a rare instance of COVID-19-associated acute long-segment transverse myelitis complicated by pulmonary embolism (PE) and lower extremity deep vein thrombosis (DVT). Although the three common autoimmune central nervous system disease antibodies (AQP4-IgG, MOG-IgG, and GFAP antibody) were all negative, the temporal association, clinical presentation, and laboratory findings strongly support the role of COVID-19 as the etiology. The intrinsic connection is not coincidental but rather untangles the complexity of the pathogenic mechanisms of the SARS-CoV-2 virus. Our findings indicate that while isolated reports of COVID-19-associated myelitis or COVID-19-associated thrombotic events exist, cases where both conditions present concurrently as primary and core manifestations are exceedingly rare. This underscores the complexity of multi-system involvement in COVID-19, making this case particularly valuable for cautionary and educational purposes.

During the diagnostic and treatment process, we also conducted a differential diagnosis with three common autoimmune central nervous system diseases and other types of myelitis: 1. Neuromyelitis Optica spectrum disorder (NMOSD): Although this patient presented with LETM, they tested negative for AQP4-IgG and showed no signs of optic neuritis, so typical NMOSD can be largely ruled out. 2. MOG antibody-associated disease (MOGAD): MOG-IgG was negative, and the clinical presentation was not consistent with this diagnosis (12). 3. Multiple sclerosis (MS): The extent of spinal cord involvement was too extensive (spanning more than three vertebral segments), which does not align with the typical presentation of MS (13). 4. Infectious myelitis: Cerebrospinal fluid (CSF) microbiological testing was negative, and there was no evidence of infection by other pathogens. 5. Spinal vascular disease: MRI findings are inconsistent with typical spinal cord infarction or hemorrhage, and the disease course is relatively slow. 6. Paraneoplastic myelitis: No history or evidence of a tumor, and antibody tests are negative.

In this case, the patient cerebrospinal fluid showed mixed lymphocytic and polymorphonuclear cell inflammation (50% each) and mildly elevated protein levels, consistent with changes seen in post-infectious or autoimmune myelitis (14). Although antibody testing was negative, this is not rare in COVID-19-associated myelitis, suggesting that the underlying immune mechanisms may differ from those of typical NMOSD or MOGAD (15). The early predominance of polymorphonuclear cells suggests intense acute inflammation, which rapidly transitions to a lymphocyte/monocyte predominance, consistent with an immune-mediated disease pattern rather than a typical bacterial infection (16). Elevated CSF protein levels reflect blood-brain barrier disruption and intrathecal immunoglobulin synthesis, serving as direct evidence of immune-inflammatory activity within the CNS and supporting a demyelinating or autoimmune etiology. The absence of detectable autoimmune antibodies ruled out the most common idiopathic inflammatory CNS disorders, such as neuromyelitis optica spectrum disorder (NMOSD) and myelin oligodendrocyte glycoprotein antibody-associated disease (MOGAD), thereby suggesting a diagnosis of an “antibody-negative, post-infectiously triggered specific autoimmune syndrome.” The patient's laboratory profile demonstrated a pattern indicative of multi-system involvement. Systemic inflammatory markers, including leukocytosis and elevated C-reactive protein (CRP) and erythrocyte sedimentation rate (ESR), were present alongside acute-phase reactants such as hypertriglyceridemia, and indicators of tissue damage, including hyperuricemia and elevated creatine kinase. These findings are consistent with a systemic inflammatory response syndrome triggered by SARS-CoV-2 infection. Hyperlactatemia suggested tissue hypoperfusion, potentially related to concurrent pulmonary embolism and circulatory dysfunction. The CSF exhibited inflammatory changes characterized by elevated protein and a mixed cellular reaction, yet all tested common CNS autoimmune antibodies were negative. This supports the diagnosis of COVID-19 as the precipitating factor for an antibody-negative acute immune-mediated myelitis. Mild elevations in liver enzymes and urinary protein likely reflect multi-organ manifestations of systemic endotheliosis and microcirculatory dysfunction.

The pathogenesis of COVID-19-related neurological complications has not yet been fully elucidated; the pathogenesis of such cases may involve an interaction between virus-induced immune dysregulation, endothelial inflammation, and a hypercoagulable state (17). This comorbidity pattern may reveal the severity of multisystem, sequential damage caused by SARS-CoV-2 infection and serves as an extreme example of the complex systemic manifestations of “long COVID” (Post-COVID-19 Condition). COVID-19 infection can induce a hypercoagulable state through various mechanisms, increasing the risk of thromboembolic events (18). We consider that the possible pathophysiological mechanisms may include: The pathophysiology of COVID-19-associated neurological complications remains incompletely understood and may involve the following mechanisms:

1. Initial Trigger: SARS-CoV-2 infection serves as the initiating event: The viral Spike protein binds to the human angiotensin-converting enzyme 2 (ACE2) receptor, which is widely expressed on alveolar epithelial cells, vascular endothelial cells, and within the nervous system (19). Although the patient in this case presented without typical respiratory symptoms, a positive nucleic acid test indicated an asymptomatic or presymptomatic infection, during which the virus can still trigger a robust systemic immune response.
2. Immune System Hyperactivation and “Cytokine Storm”: Viral infections trigger the activation of both innate and adaptive immune responses, leading to the release of a large quantity of inflammatory cytokines (e.g., IL-6, IL-1β, TNF-α) (20). The marked elevation of neutrophils and the sharp rise in D-dimer levels observed in this patient during the initial phase precisely reflect a systemic inflammatory response and a hypercoagulable state. These cytokines disrupt the blood-nerve barrier (BNB) and the blood-brain barrier (BBB), permitting the infiltration of autoreactive T cells and B cells into the central nervous system (21).
3. Molecular Mimicry and Autoimmune Attack: viral antigens may exhibit structural similarities to human self-antigens (e.g., neural proteins), a phenomenon known as molecular mimicry. This can induce a cross-reactive immune response that attacks spinal cord tissue, resulting in acute myelitis (22). In this patient, the absence of specific antibodies ruled out common idiopathic disorders such as neuromyelitis optica spectrum disorder (NMOSD) and myelin oligodendrocyte glycoprotein antibody-associated disease (MOGAD). The clinical presentation was more consistent with a post-infectious, antibody-negative autoimmune myelitis. The sign of “dissociated sensory loss” (loss of pain and temperature sensation with preserved proprioception and light touch), resulting from anterior spinal cord involvement, closely corresponds to the manifestations of ischemic injury within the territory supplied by the anterior spinal artery.
4. Endotheliosis, Hypercoagulable State, and Thrombosis: In this patient, the D-dimer level was significantly elevated, reaching a peak of 1,732.0 ng/mL, before gradually decreasing, thereby demonstrating the efficacy of anticoagulant therapy. The concurrent presence of PE and lower extremity DVT indicated the necessity for aggressive anticoagulation. Patients with COVID-19 have a significantly increased risk of thromboembolism. The potential mechanisms are considered to include: Vascular endothelial injury: Direct viral infection of endothelial cells and the cytokine storm led to extensive endothelial cell damage and activation (endotheliosis) (23). Activation of the coagulation system: Activated endothelial cells release von Willebrand factor (vWF), downregulate thrombomodulin, and express tissue factor, creating a procoagulant state (24). Inflammatory cytokines activate the coagulation cascade, elevating fibrinogen and D-dimer. Platelet activation is also induced by the virus, contributing to the formation of microthrombi. Additionally, blood stasis, exacerbated by myelitis-induced paralysis, further elevates the risk of venous thrombosis in the lower extremities (25). The markedly elevated D-dimer level and the rapid development of DVT and PE in this patient were direct consequences of this process.
5. The Association Between Myelitis and Thrombosis: In this case, the concurrent occurrence of acute long-segment transverse myelitis and pulmonary embolism was not coincidental but rather reflected a single pathophysiological pathway centered on virus-induced endothelial damage and an excessive inflammatory response. SARS-CoV-2 binds to ACE2 receptors widely distributed on the vascular endothelium and the blood-brain barrier, triggering endotheliosis and a cytokine storm, while simultaneously pushing the body into a procoagulant state and igniting neuroinflammation (20). The resulting endothelial dysfunction not only compromises vascular integrity but also activates an immune-thrombotic cascade, including the release of neutrophil extracellular traps (NETs) and platelet activation—rendering venous thromboembolism more likely to occur (26). Concurrently, the same inflammatory environment, coupled with potential molecular mimicry mechanisms, can breach the immune privilege of the central nervous system, inducing autoimmune myelitis (27). In this patient, the rapid progression of the disease led to flaccid paralysis, which further exacerbated venous stasis (one of Virchow's three criteria), in turn amplifying the risk of thrombosis. It can be said that these two complications are not independent of one another but rather form a vicious cycle: neuroinflammation and immune thrombogenesis mutually reinforce and exacerbate each other, aptly illustrating the systemic nature of COVID-19-associated immune-mediated injury.

## 4 Conclusion

This case report presents a rare instance of COVID-19-associated acute long-segment transverse myelitis complicated by pulmonary embolism and lower extremity deep vein thrombosis. The pathophysiology may involve virus-induced intense systemic immune-inflammatory responses and a hypercoagulable state, which respectively attack the spinal cord and the vascular system. Subsequent paralysis-induced blood stasis further precipitated the thrombotic events. A dual-pronged approach was therefore essential: employing immunomodulatory/suppressive agents (e.g., high-dose steroids) to control neuroinflammation while implementing anticoagulation for thrombotic complications, with vigilant monitoring for the potentially increased bleeding risk.

## Data Availability

The original contributions presented in the study are included in the article/Supplementary Material, further inquiries can be directed to the corresponding authors.

## References

[1] Acharya A Kevadiya BD Gendelman HE Byrareddy SN. SARS-CoV-2 infection leads to neurological dysfunction. J Neuroimmune Pharmacol. (2020) 15:167–73. 10.1007/s11481-020-09924-9 32447746 PMC7244399

[2] Puelles VG Lütgehetmann M Lindenmeyer MT Sperhake JP Wong MN Allweiss L Multiorgan and renal tropism of SARS-CoV-2. N Engl J Med. (2020) 383(6):590–2. 10.1056/NEJMc2011400 32402155 PMC7240771

[3] Law N Chan J Kelly C Auffermann WF Dunn DP. Incidence of pulmonary embolism in COVID-19 infection in the ED: ancestral, Delta, Omicron variants and vaccines. Emerg Radiol. (2022) 29:625–9. 10.1007/s10140-022-02039-z 35446000 PMC9022402

[4] Katsoularis I Fonseca-Rodríguez O Farrington P Jerndal H Lundevaller EH Sund M Risks of deep vein thrombosis, pulmonary embolism, and bleeding after COVID-19: nationwide self-controlled cases series and matched cohort study. Br Med J. (2022) 377:e069590. 10.1136/bmj-2021-069590 35387772 PMC8984137

[5] Dutch COVID & Thrombosis Coalition, Kaptein FHJ Stals MAM Grootenboers M Braken SJE Burggraaf JLI Incidence of thrombotic complications and overall survival in hospitalized patients with COVID-19 in the second and first wave. Thromb Res. (2021) 199:143–8. 10.1016/j.thromres.2020.12.019 33535120 PMC7832218

[6] Tudoran C Tudoran M Abu-Awwad A Cut TG Voiță-Mekereș F. Spontaneous hematomas and deep vein thrombosis during the recovery from a SARS-CoV-2 infection: case report and literature review. Medicina (Kaunas). (2022) 58(2):230. 10.3390/medicina58020230 35208553 PMC8878215

[7] Helms J Tacquard C Severac F Leonard-Lorant I Ohana M Delabranche X High risk of thrombosis in patients with severe SARS-CoV-2 infection: a multicenter prospective cohort study. Intensive Care Med. (2020) 46(6):1089–98. 10.1007/s00134-020-06062-x 32367170 PMC7197634

[8] Ma J Xi J Sun Y Yang S Li H Zhao C. Acute myelitis following COVID-19 infection (with 5 case reports and a literature review). Chin J Clin Neurosci. (2023) 31(04):418–24, 431.

[9] Román GC Gracia F Torres A Palacios A Gracia K Harris D. Acute transverse myelitis (ATM): clinical review of 43 patients with COVID-19-associated ATM and 3 post-vaccination ATM serious adverse events with the ChAdOx1 nCoV-19 vaccine (AZD1222). Front Immunol. (2021) 12:653786. 10.3389/fimmu.2021.653786 33981305 PMC8107358

[10] Ahmad F Kannan M Ansari AW. Role of SARS-CoV-2-induced cytokines and growth factors in coagulopathy and thromboembolism. Cytokine Growth Factor Rev. (2022) 63:58–68. 10.1016/j.cytogfr.2021.10.007 34750061 PMC8541834

[11] Rao SN Manissero D Steele VR Pareja J. A systematic review of the clinical utility of cycle threshold values in the context of COVID-19. Infect Dis Ther. (2020) 9(3):573–86. 10.1007/s40121-020-00324-3 32725536 PMC7386165

[12] Filippatou AG Said Y HaiwenVasileiou C Vasileiou ES Ahmadi G Sotirchos ES. Validation of the international MOGAD panel proposed criteria: a single-centre US study. J Neurol Neurosurg Psychiatry. (2024) 95(9):870–3. 10.1136/jnnp-2023-333227 38569875 PMC11330367

[13] McGinley MP Goldschmidt CH Rae-Grant AD. Diagnosis and treatment of multiple sclerosis: a review. JAMA. (2021) 325(8):765–79. 10.1001/jama.2020.26858 33620411

[14] Douglas AG Xu DJ Shah MP. Approach to myelopathy and myelitis. Neurol Clin. (2022) 40(1):133–56. 10.1016/j.ncl.2021.08.009 34798966

[15] Uzawa A Oertel FC Mori M Paul F Kuwabara S. NMOSD and MOGAD: an evolving disease spectrum. Nat Rev Neurol. (2024) 20(10):602–19. 10.1038/s41582-024-01014-1 39271964

[16] Domingues RB Leite FBVM Senne C. Cerebrospinal fluid analysis in patients with COVID-19-associated central nervous system manifestations: a systematic review. Arq Neuropsiquiatr. (2022) 80(3):296–305. 10.1590/0004-282x-anp-2021-0117 35239818 PMC9648929

[17] AlKetbi R AlNuaimi D AlMulla M AlTalai N Samir M Kumar N Acute myelitis as a neurological complication of COVID-19: a case report and MRI findings. Radiol Case Rep. (2020) 15(9):1591–5. 10.1016/j.radcr.2020.06.001 32685076 PMC7275163

[18] Flaumenhaft R Enjyoji K Schmaier AA. Vasculopathy in COVID-19. Blood. (2022) 140(3):222–35. 10.1182/blood.2021012250 34986238 PMC8736280

[19] Ashraf UM Abokor AA Edwards JM Waigi EW Royfman RS Hasan SA SARS-CoV-2, ACE2 expression, and systemic organ invasion. Physiol Genomics. (2021) 53(2):51–60. 10.1152/physiolgenomics.00087.2020 33275540 PMC7900915

[20] Mehta P McAuley DF Brown M Sanchez E Tattersall RS Manson JJ COVID-19: consider cytokine storm syndromes and immunosuppression. Lancet. (2020) 395(10229):1033–4. 10.1016/S0140-6736(20)30628-0 32192578 PMC7270045

[21] Suprewicz Ł Fiedoruk K Czarnowska A Sadowski M Strzelecka A Galie PA Blood-brain barrier function in response to SARS-CoV-2 and its spike protein. Neurol Neurochir Pol. (2023) 57(1):14–25. 10.5603/PJNNS.a2023.0014 36810757

[22] Pedebos C Khalid S. Simulations of the spike: molecular dynamics and SARS-CoV-2. Nat Rev Microbiol. (2022) 20(4):192. 10.1038/s41579-022-00699-9 35121799 PMC8815016

[23] Varga Z Flammer AJ Steiger P Haberecker M Andermatt R Zinkernagel AS Endothelial cell infection and endotheliitis in COVID-19. Lancet. (2020) 395(10234):1417–8. 10.1016/S0140-6736(20)30937-5 32325026 PMC7172722

[24] Abou-Ismail MY Diamond A Kapoor S Arafah Y Nayak L. The hypercoagulable state in COVID-19: incidence, pathophysiology, and management. Thromb Res. (2020) 194:101–15. 10.1016/j.thromres.2020.06.029 32788101 PMC7305763

[25] Li S Wang H Shao Q. The central role of neutrophil extracellular traps (NETs) and by-products in COVID-19 related pulmonary thrombosis. Immun Inflamm Dis. (2023) 11(8):e949. 10.1002/iid3.949 37647446 PMC10461423

[26] Zuo Y Yalavarthi S Shi H Gockman K Zuo M Madison JA Neutrophil extracellular traps in COVID-19. JCI Insight. (2020) 5(11):e138999. 10.1172/jci.insight.138999 32329756 PMC7308057

[27] Paterson RW Brown RL Benjamin L Nortley R Wiethoff S Bharucha T The emerging spectrum of COVID-19 neurology: clinical, radiological and laboratory findings. Brain. (2020) 143(10):3104–20. 10.1093/brain/awaa240 32637987 PMC7454352

